# Cytotoxic Activity of *Boswellia serrata* Roxb. Essential Oil and Acetyl-11-Keto-β-Boswellic Acid (AKBA) on Hepatocellular Carcinoma Cells: In Vitro and In Silico Study

**DOI:** 10.3390/ijms27135978

**Published:** 2026-07-03

**Authors:** Francisco Javier Alarcon-Aguilar, Diana Laura Torres-Chacón, Alfredo Suárez-Alonso, Samuel Enoch Estrada-Soto, Luis Enrique Gómez-Quiroz, José Luís Eduardo Flores Sáenz, Elisa Vega Ávila, Gerardo Blancas Flores, Abraham Giacoman Martínez, Beatriz Mora Ramiro, Julio César Almanza-Pérez

**Affiliations:** 1 Departamento de Ciencias de la Salud, División de Ciencias Biológicas y de la Salud, Universidad Autónoma Metropolitana—Iztapalapa, Ciudad de México 09340, Mexico; aaaf@xanum.uam.mx (F.J.A.-A.); ipn_2nv4@hotmail.com (A.S.-A.); legomezq@correo.uam.mx (L.E.G.-Q.); luisflosaenz@gmail.com (J.L.E.F.S.); vega@xanum.uam.mx (E.V.Á.); gera@xanum.uam.mx (G.B.F.); 2 Posgrado en Biología Experimental, División de Ciencias Biológicas y de la Salud, Universidad Autónoma Metropolitana—Iztapalapa, Ciudad de México 09340, Mexico; dianat.chacon@gmail.com; 3 Facultad de Farmacia, Universidad Autónoma del Estado de Morelos, Cuernacava 62209, Estado de Morelos, Mexico; enoch@uaem.mx (S.E.E.-S.); beatriz.mo.ramiro@gmail.com (B.M.R.); 4 Centro de Investigación Biomédica del Sur, Instituto Mexicano del Seguro Social, Xochitepec 62790, Estado de Morelos, Mexico; abraham.giacoman@imss.gob.mx

**Keywords:** hepatocellular carcinoma, *Boswellia serrata*, AKBA, longifolene, 5-LO, natural products

## Abstract

Hepatocellular carcinoma is one of the most aggressive malignancies worldwide, with limited therapeutic options. *Boswellia serrata* Roxb., an Indian medicinal tree, produces a resin rich in essential oil and boswellic acids, particularly acetyl-11-keto-β-boswellic acid (AKBA), with demonstrated antiproliferative and pro-apoptotic activities. This study investigated the cytotoxic effects of *B. serrata* essential oil and AKBA on hepatocarcinoma Huh-7 cells in both monolayer and three-dimensional spheroid cultures and characterized the underlying molecular targets. Essential oil was extracted and analyzed by gas chromatography-mass spectrometry (GC-MS). Cytotoxicity was assessed using the cell counting kit-8 (CCK-8). Three-dimensional spheroid cultures were also established to evaluate anti-tumoral potential. Expression of cyclin D1, cyclin-dependent of kinase 4 (CDK4) (cyclin-dependent kinase inhibitor 1A (p21), E-cadherin, (alpha fetoprotein) AFP, epithelial cell adhesion molecule (EpCAM), Myeloid cell leukemia-1 (Mcl-1), and caspase-3 was analyzed by western blot. In addition, an in silico analysis was performed on the main constituents of *B. serrata* essential oil targeting 5-lipoxygenase (5LO). The results showed cytotoxic effects, with AKBA exhibiting greater potency than the essential oil. Cytotoxicity was associated with caspase-3-mediated apoptosis, with minimal effects on cell cycle and epithelial–mesenchymal transition markers. The in silico analysis predicted that some compounds may act as competitive inhibitors of the 5LO at the catalytic site and partially activate pro-apoptotic pathways. These data support the potential of *B. serrata*-derived compounds as novel anti-hepatocarcinoma agents, with AKBA and longifolene as leads for further preclinical and clinical research.

## 1. Introduction

Hepatocellular carcinoma (HCC) represents a worldwide health challenge, ranking as the sixth most common cancer and the third leading cause of cancer-related mortality [1]. In Mexico, HCC is tenth in incidence and sixth in mortality, with low five-year survival rates [1,2]. The disease occurs through progressive hepatic injury, cirrhosis, and malignant transformation, often following chronic hepatitis B or C infection, alcohol-related liver disease, or metabolic syndrome [2,3]. Current treatment alternatives, including surgical resection, liver transplantation, hepatic arterial chemoembolization, sorafenib, lenvatinib, atezolizumab, bevacizumab, and, more recently, immunotherapy, demonstrate variable efficacy and are frequently constrained by toxicity, drug resistance, or advanced-stage disease [3,4]. The emergence of acquired resistance to tyrosine kinase inhibitors, driven by the selection of cells with activating mutations in growth-signaling pathways, constitutes a major clinical obstacle [3]. The alternative of immunotherapy addresses this resistance by inhibiting immune checkpoint interactions, such as programmed death-1 (PD-1) and programmed death-ligand 1 (PD-L1); however, access to these therapies is often expensive [4]. Therefore, there is an urgent need to identify novel therapeutic agents for malignant hepatocytes [5].

*Boswellia serrata* Roxb. (Burseraceae), an Indian medicinal tree whose resin is known as frankincense, has been utilized for centuries in traditional Ayurvedic medicine as an anti-inflammatory agent and for the treatment of rheumatoid arthritis [6]. The resin has demonstrated hepatoprotective, cardioprotective, and neuroprotective properties in preclinical studies [7,8,9]. It contains volatile monoterpenes and sesquiterpenes (comprising the essential oil fraction), as well as non-volatile pentacyclic triterpenes (boswellic acids), which constitute 30 to 65% of the resin [10,11].

The acetyl-11-keto-β-boswellic acid (AKBA) is recognized as the predominant pharmacologically active component [11,12]. AKBA modulates inflammatory signaling by inhibiting NF-κB and pro-inflammatory cytokine production [12]. Furthermore, AKBA has demonstrated pro-apoptotic activity against various cancer types in vitro, including hepatocellular carcinoma, via caspase-dependent and -independent pathways [12]. In HepG2 hepatocarcinoma cells, AKBA induced caspase-8-mediated apoptosis [13]. Additionally, *B. serrata* extracts and doxorubicin exhibited synergistic cytotoxicity [14].

An enzyme that plays a fundamental role in the induction of apoptosis is the 5-lipoxygenase (5LO), which catalyzes the synthesis of inflammatory mediators [15]. 5LO can be regulated by the 5LO activator protein (FLAP). There is also evidence that inflammatory factors increase 5LO expression and activity [16]. In this context, AKBA is a non-competitive inhibitor of 5LO, acting at an allosteric site located between the catalytic and membrane-binding domains [13]. Inhibition of FLAP by MK-866 consequently inhibits 5LO, induces apoptosis in gastric cancer cells by activating caspase 3, and upregulates cyclin-dependent kinase inhibitor 1B (p27kip1) and Bc l-2-associated X protein (Bax). This occurs without altering the expression of tumor protein p53, cyclin-dependent kinase inhibitor 1A (p21), or B-cell lymphoma 2 (bcl-2) [17]. 5LO promotes the production of 5 and 12 12-hydroxyeicosatetraenoic acids (5-HETE and 12-HETE, respectively), and leukotriene B4 (LTB4), which promote mitogen-activated protein kinase kinase/Extracellular signal-regulated kinase (MEK/ERK) phosphorylation, a pathway known to prevent apoptosis. It is also known that 5LO products activate the phosphoinositide 3-kinase/protein kinase B (PI3K/AKT) cascade, which has the same effect on apoptosis [18]. Thus, 5LO inhibition causes an imbalance between anti- and pro-apoptotic proteins, favoring the latter. Previous studies on *B. serrata* have primarily focused on crude resin extracts or isolated boswellic acids and have limited direct comparisons between the essential oil fraction and pure AKBA under standardized cellular conditions.

Although advances such as the integration of some research methodologies have been achieved, comprehensive analyses incorporating cytotoxicity profiling, three-dimensional culture models, and detailed molecular mechanistic evaluation remain scarce. In addition, the study of the contributions of monoterpene and triterpene components in the essential oil to overall cytotoxicity is also mandatory. The present study was designed to characterize the cytotoxic activities of *B. serrata* essential oil and AKBA against human hepatocarcinoma Huh-7 cells using complementary in vitro and in silico models, to clarify underlying molecular mechanisms, and to provide a basis for future drug development and preclinical and clinical evaluation in HCC models.

## 2. Results

### 2.1. Phytochemical Characterization of B. serrata Essential Oil

Direct steam distillation of *B. serrata* resin yielded 5% (*w*/*w*) essential oil with characteristic organoleptic properties: an amber color, a sweet-citrus odor, a viscous liquid appearance, and a greasy texture. Gas chromatography-mass spectrometry (GC-MS) analysis identified 21 different chemical constituents, accounting for 98.7% of the analyzed oil (Table 1). The predominant components were: terpinen-4-ol (7.81%), p-cymen-8-ol (2.00%), α-terpineol (2.96%), isoterpinolene (2.96%), and terpinolene (2.96%), with additional monoterpene and sesquiterpene constituents including linalool, β-thujene, p-cymene, and trace amounts of elemicin, valencene, and longifolene. These volatile constituents are consistent with the terpene-rich composition documented in preceding investigations of *B. serrata* essential oils [19,20,21].

### 2.2. Effects of B. serrata Essential Oil and AKBA on Huh-7 Cell Proliferation

**Figure 1:**
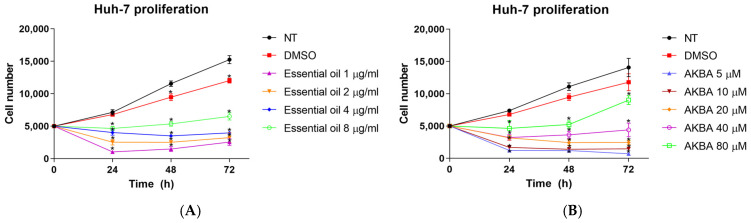
Effects of *B. serrata* essential oil (**A**) and acetyl-11-keto-β-boswellic acid (AKBA) (**B**) on Huh-7 cell proliferation. *n* = 4; * *p* < 0.05. NT = No treatment. DMSO = Dimethyl sulfoxide (vehicle control).

Treatment of Huh-7 cells with the *B. serrata* essential oil demonstrated dose- and time-dependent inhibition of cell proliferation (Figure 1). At 24 h post-treatment with essential oil, concentrations of 1 and 2 μg/mL produced approximately 35% and 40% reductions in cell viability, respectively, which progressively increased to 80% and 85% inhibition by 72 h (Figure 1A). Concentrations of 4 μg/mL and 8 μg/mL showed dose-limiting effects at 24 h (42% and 50% inhibition, respectively), with relative stabilization of the response by 72 h, implying possible plateau or compensatory responses at higher doses. The IC_50_ of the essential oil was approximately 1 μg/mL after 72 h of exposure. Vehicle control produced modest growth inhibition (almost 10% reduction in viability) compared with untreated controls, but the difference was not statistically significant. The essential oil displayed superior selectivity to untreated controls but lower potency than purified AKBA, as shown below.

AKBA treatment produced more pronounced cytotoxic effects than the essential oil across the tested concentration range (Figure 1B). At 24 h, concentrations of 5 μM and 10 μM induced approximately 50% and 55% reductions in cell viability, respectively. By 72 h, these concentrations reduced cell viability to approximately 10% and 5%, respectively, representing 90% and 95% inhibition. Concentrations of 20 μM and 40 μM showed more rapid effects, with maximal inhibition achieved by 48 h. The highest concentration tested (80 μM) showed partial recovery in viability by 72 h (approximately 20% of the control). The IC_50_ for AKBA was estimated at approximately 5 μM at 72 h. These results show that AKBA is more potent than the whole essential oil.

### 2.3. Effect on Three-Dimensional Spheroid Architecture and Viability

**Figure 2:**
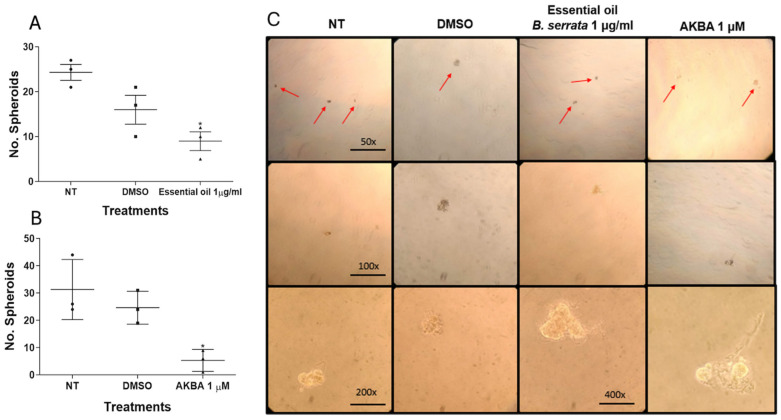
Effect of *Boswellia serrata* essential oil and AKBA on spheroid formation. Quantification of the number of spheroids after treatment with *B. serrata* essential oil 1 µg/ml (**A**) and with AKBA 1 µM (**B**). Representative photomicrographs of the cultures under the different treatments (**C**); red arrows indicate the presence of spheroids. Values were presented as means ± SEM. *N* = 4, * *p* ≤ 0.05. NT = No Treatment. DMSO = Vehicle control.

To evaluate cytotoxic effects in a more physiologically relevant microenvironment, we assessed the impact of treatment on three-dimensional Huh-7 spheroid formation and viability (Figure 2).

Untreated control spheroids grew progressively over 5 days of culture, reaching mean diameters of 150–200 μm and preserving consistent spheroid numbers (approximately 100–120 per well). Treatment with 1 μg/mL *B. serrata* essential oil reduced spheroid number by 65% (*p* < 0.001) and mean diameter by 58% (*p* < 0.05) relative to untreated controls following 72 h post-treatment (Figure 2A,C). Similarly, AKBA treatment at 1 μM reduced spheroid number by 72% (*p* < 0.001) and mean diameter by 68% (*p* < 0.01), manifesting superior efficacy compared with the essential oil in the spheroid context (Figure 2B,C). Vehicle control produced no significant effects on spheroid architecture or number (Figure 2C). The data indicate that both *B. serrata* essential oil and AKBA retain cytotoxic activity inside three-dimensional structures.

### 2.4. Molecular Characterization of Cell Cycle Regulatory Proteins

**Figure 3:**
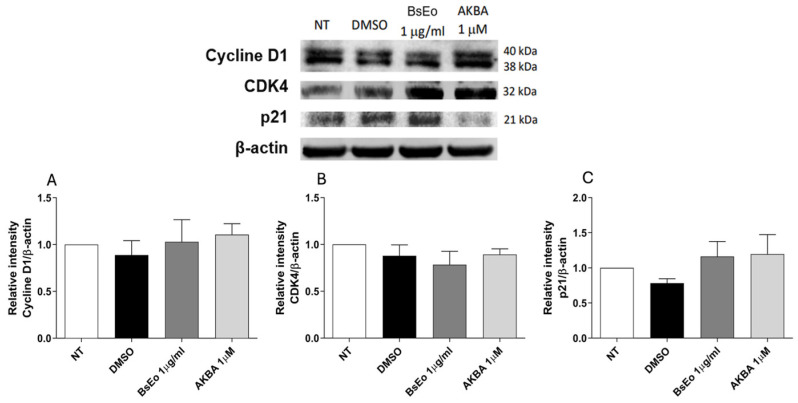
Effect of *B. serrata* essential oil (BsEo) and AKBA on expression of cell cycle checkpoint proteins: Cyclin D1 (**A**), CDK4 (**B**), and p21 (**C**). *n* = 4. NT = No treatment. DMSO = Vehicle control.

To explain the molecular mechanisms underlying the observed cytotoxic effects, we evaluated the expression of cell cycle checkpoint proteins known to be deregulated in hepatocellular carcinoma. Protein expression analysis showed that treatment with 1 μg/mL *B. serrata* essential oil or 1 μM AKBA for 72 h produced no significant changes in expression of cyclin D1, CDK4, or p21 relative to untreated controls (Figure 3). Cyclin D1 expression remained relatively stable, as did CDK4 and p21 protein levels.

### 2.5. Evaluation of Epithelial/Mesenchymal Transition (EMT) Markers

**Figure 4:**
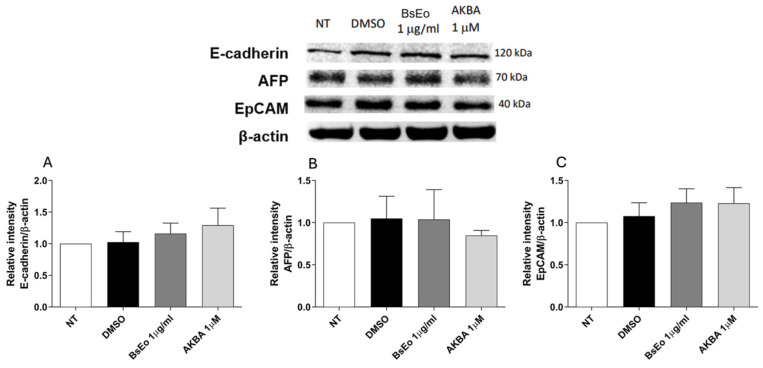
Effect of *B. serrata* essential oil (BsEo) and AKBA on expression of epithelial differentiation and epitelian mesenquimal transition (EMT)-associated markers: E-cadherin (**A**), EpCAM (**B**), and AFP (**C**). *n* = 4. NT = No treatment. DMSO = Vehicle control.

Expression of epithelial differentiation and EMT-associated markers was assessed as potential indicators of effects on metastatic potential and stemness (Figure 4). Treatment with either essential oil or AKBA produced minimal changes in E-cadherin expression, with slightly increased expression in AKBA-treated cells (15% increase) that did not reach statistical significance. Similarly, the expression of EpCAM and AFP, markers of a hepatic stem cell-like differentiation state, remained significantly unchanged across treatment conditions.

### 2.6. Activation of Apoptotic Pathways

**Figure 5:**
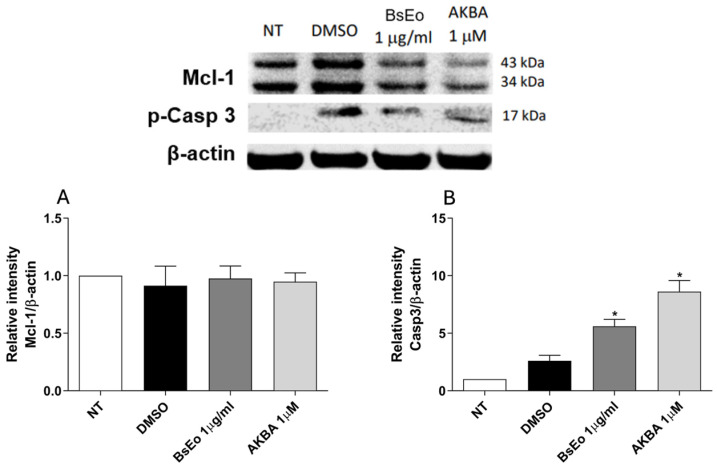
Effect of *B. serrata* essential oil (BsEo) and AKBA on activation of apoptotic pathways: Mcl-1 (**A**) and Caspase-3 (**B**). *n* = 4; * *p* < 0.05. NT = No treatment. DMSO = Vehicle control.

In contrast to the minimal effects on cell cycle and EMT markers, treatment with 1 μg/mL *B. serrata* essential oil, particularly with 1 μM AKBA, activated pro-apoptotic signaling. Protein expression analysis showed upregulation of caspase-3 protein following treatment (Figure 5). Quantification via densitometry revealed that AKBA treatment induced approximately a 2.8-fold increase in cleaved caspase-3 levels relative to untreated controls (*p* < 0.05), while *B. serrata* essential oil induced approximately 1.9-fold increase (*p* < 0.05). Concurrent with caspase-3 activation, Mcl-1 expression did not change significantly. The predominant apoptotic signature was therefore activation of executioner caspase-3, consistent with engagement of the caspase-dependent programmed cell death pathway.

### 2.7. Molecular Docking Simulations

**Figure 6:**
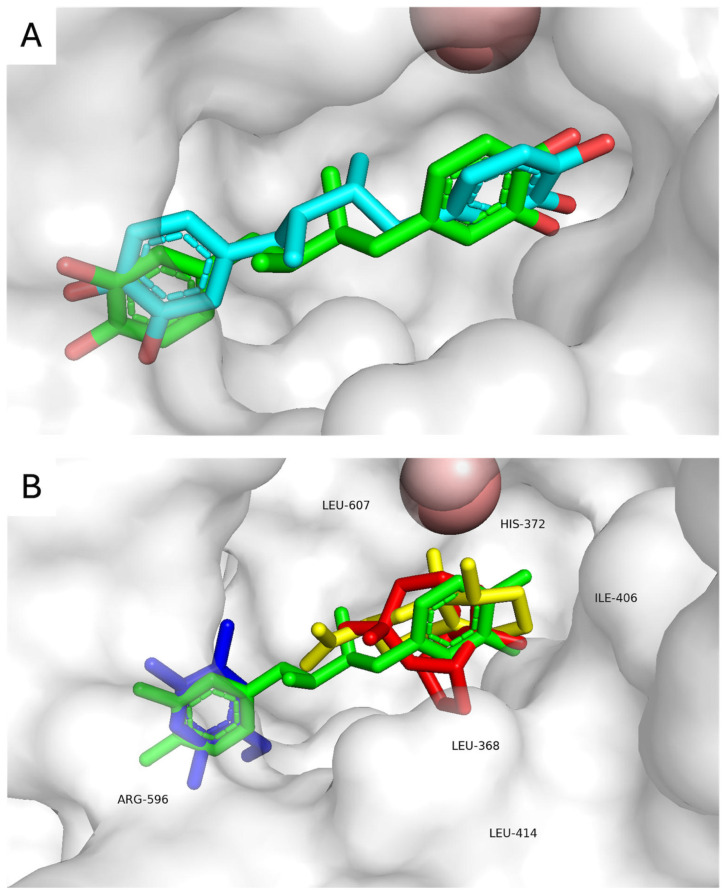
Three-dimensional superposition of the results of molecular docking simulations at the 5-lipoxygenase (5LO) catalytic site. The brown sphere is the catalytic iron. (**A**) Re-docking analysis. The green hydrocarbon chain represents the crystallographic pose of NDGA; the cyan chain is the pose predicted by docking. (**B**) Overlap of the poses for the best three predicted binding energies. Red for longifolene, yellow for valencene, and blue for 2-Ethyl-4,5-dimethylphenol. The crystallographic pose of NDGA is shown as a reference (green).

The re-docking simulation of AKBA at the allosteric inhibition site failed to reproduce the crystallographic pose of the 5LO. The calculated root-mean-square deviation (RMSD) value for the simulated pose was 8.853 Å, while the predicted binding energy for this pose was −9.12 kcal/mol. In contrast, the nor-dihydro-guaretic acid (NDGA) pose was successfully reproduced at the catalytic site of 5LO, with an RMSD of 1.643 Å relative to the crystallographic pose (Figure 6), and the predicted binding energy was −8.3 kcal/mol. The compounds in the *B. serrata* essential oil showed predicted binding energies ranging from −6.7 to −5.4 kcal/mol (Table 2). Collectively, the compounds occupied the same space as NDGA (Figure 6).

**Figure 7:**
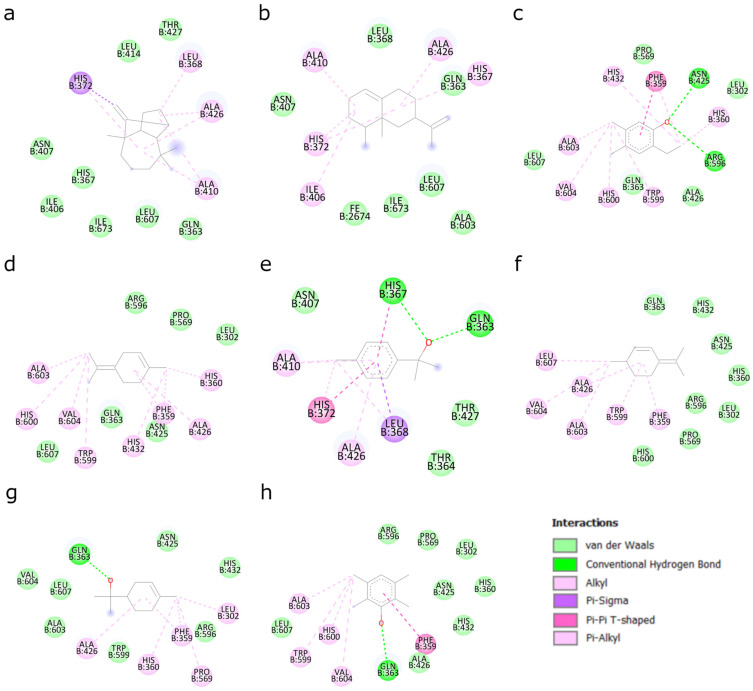
Two-dimensional representation of the predicted molecular docking interactions of *B. serrata* essential oil compounds at the 5LO enzyme catalytic site. They are in ascending order of binding energy, from left to right and top to bottom. Here we represent the best eight binding energy compounds: (**a**) longifolene, (**b**) valencene, (**c**) 2-ethyl-4,5-dimethylphenol, (**d**) terpinolene, (**e**) p-cymen-8-ol, (**f**) isoterpinolene, (**g**) α-terpineol, and (**h**) durenol. The dashed lines indicate the interaction between the ligand and the amino acid residue. The type of interaction is specified by the color code.

Figure 7 shows a two-dimensional representation of the predicted molecular docking interactions of *B. serrata* essential oil compounds at the 5LO enzyme catalytic site. Longifolene binds in the vicinity of Leu414 with an energy prediction of −6.7 kcal/mol (Table 2). This amino acid residue is important for substrate positioning during the catalytic event, along with Leu368, Leu607, and Ile406, which were also predicted to interact with the compounds (Figure 7). Arg596 appeared frequently in molecular docking simulations and was identified as one interaction site with NDGA. His372 also appeared several times in the interactions (Figure 7).

## 3. Discussion

*B. serrata* essential oil and its constituent boswellic acid, AKBA, exert cytotoxic effects on human hepatocellular carcinoma Huh-7 cells by activating caspase-dependent apoptosis. The GC-MS characterization revealed a volatile monoterpene-enriched profile consistent with earlier analyses of *B. serrata* essential oils, with predominant constituents including terpinen-4-ol, α-terpineol, p-cymen-8-ol, and terpinolene [20,21]. While the boswellic acids (pentacyclic triterpenes) responsible for anti-inflammatory and anti-cancer activities are non-volatile and therefore not present in the essential oil fraction, the identified monoterpenes possess documented biological activities. Specifically, α-terpineol has demonstrated pro-apoptotic activity against cancer cells through multiple mechanisms, including disruption of mitochondrial membrane potential and caspase activation [22]. Linalool, also identified in the oil, exhibits anti-proliferative properties in hepatocarcinoma cells through p53-dependent pathways [23]. These monoterpene constituents may contribute in an additive or synergistic manner to the observed cytotoxic phenotype, accounting for the approximately 5–10-fold lower potency of the essential oil relative to purified AKBA.

The 5% yield of essential oil from resin processing is consistent with previously reported values for the volatile fraction recovery of *B. serrata* [21]. AKBA is a single, highly optimized bioactive molecule, whereas the essential oil comprises a complex mixture of constituents with diverse bioactivities and potencies. The identification of monoterpenes with documented pro-apoptotic activities supports the strategy of molecular fractionation and single-compound isolation for drug development. Simultaneously, crude botanical extracts and essential oils may serve as multi-component therapeutics that interact with multiple molecular targets [24]. Additional structure-activity relationship (SAR) studies using individual boswellic acids and essential oil constituents, both in isolation and in combination, are necessary to clarify the relative contributions of specific compounds. These aspects were addressed in the present research, and the results will be discussed subsequently.

Three-dimensional spheroid cultures deliver several benefits over monolayer approaches for evaluating cancer therapeutics. Spheroids recapitulate aspects of solid tumor biology, including hypoxic core regions, reduced metabolic activity, altered cell cycle distribution, and increased apoptotic sensitivity [25]. In this experiment, mature spheroids were treated with selected concentrations of essential oil (IC50 1 μg/mL) and AKBA (sub-IC50 1 μM). In the initial studies, mature spheroids were first treated with AKBA at 5 μM; however, the results with this concentration prevented the spheroid formation (Supplementary Materials). Therefore, for all the posterior experiments (spheroid and Western blot assays), it was necessary to adjust to the sub-IC50 concentration of 1 μM. This concentration decreased spheroid number and mean diameter relative to untreated controls, indicating that spheroids are highly sensitive to AKBA at lower concentrations.

The observation that both *B. serrata* essential oil and AKBA retained substantial cytotoxic activity in spheroid cultures is therefore encouraging for subsequent in vivo evaluation. The relative preservation of potency in the more challenging 3D environment suggests that these compounds may show sufficient potency to overcome the increased drug resistance and metabolic stress characteristic of solid tumors [25].

The primary molecular signature of *B. serrata* and AKBA-induced cytotoxicity was activation of cleaved caspase-3, the key executioner caspase in both extrinsic (death receptor-mediated) and intrinsic (mitochondrial-mediated) apoptotic pathways [13,14,15]. The fold-increases in caspase-3 activation (1.9–2.8-fold) are consistent with prior findings in HepG2 hepatocarcinoma cells [13,14,15] and suggest the engagement of a genuine pro-apoptotic response distinct from non-specific cell damage or necrosis.

Notably, minimal changes were observed in the anti-apoptotic protein Mcl-1, a Bcl-2 family member frequently overexpressed in hepatocellular carcinoma and conferring resistance to mitochondrial permeabilization and caspase activation [26]. This observation implies that caspase-3 activation is not primarily dependent upon suppression of Mcl-1 but rather may reflect enhanced activation of pro-apoptotic BCL-2 family members (BAX, Bcl2 antagonist/killer 1 (BAK)) or engagement of the extrinsic (death receptor) pathway through upregulation of death receptor 5 (DR5) or other death receptors, mechanisms documented for boswellic acids in other cancer contexts [27].

The absence of substantial effects on Cyclin D1, CDK4, and p21 is unexpected, as cell cycle checkpoint disruption is a hallmark of both chemotherapy-induced apoptosis and natural product-mediated cell death in many cancer contexts [3,4,5,6]. This observation may be attributable to the 72 h timepoint, cellular arrest in the G1 phase, or the possibility that the Huh-7 cell line relies more on apoptotic rather than cell-cycle checkpoint mechanisms [28]. Western blot time-course studies at earlier timepoints are necessary to clarify these mechanistic questions.

Minimal changes in epithelial markers (E-cadherin) and hepatic stem cell markers (AFP, EpCAM) suggest that the cytotoxic effects do not primarily operate through forced epithelial differentiation or the suppression of EMT/stemness programs, unlike some other plant-derived anti-cancer agents [29]. This is notable given that many advanced HCCs exhibit EMT-like phenotypes and cancer stem cell enrichment, both of which are associated with enhanced chemoresistance [30]. The preservation of E-cadherin expression in surviving cells may facilitate the restoration of epithelial phenotype following transient or sub-lethal treatment or may indicate that the surviving cell population after an acute cytotoxic insult retains epithelial characteristics. Upcoming investigations employing dose escalation to non-lethal concentrations or longer treatment durations may reveal delayed effects on differentiation status.

Prior independent investigations have demonstrated that AKBA exhibits negligible cytotoxicity and preserves basal cell viability in normal human skin fibroblasts (HSF) [31] and HEK293 models [32] at micromolar ranges where malignant hepatocytes concurrently undergo massive apoptotic cascades. This marked selectivity is driven by the fact that AKBA operates as a targeted, non-competitive allosteric inhibitor of the 5-LOX/FLAP signaling axis, a pathway that is heavily upregulated in transformed cells like Huh-7 to promote survival via MEK/ERK and PI3K/AKT phosphorylation but remains mostly dormant or non-essential for the baseline survival of non-transformed somatic tissues [33].

The re-docking simulation failed to reproduce AKBA’s pose. Even Vina’s scoring function indicates poor binding energy for the native pose. This could be due to the difficulties experienced by the 6NCF structure depositors during refinement, resulting from high thermal motion values in this region [16]. In any case, we concluded that the molecular docking results of the *B. serrata* essential oil compounds at the allosteric inhibition site are not suitable for analysis.

In the 5LO catalytic site, the docking simulations reproduced the crystallographic pose of the NDGA inhibitor with an RMSD of less than 2 Å, in accordance with the general convention, thereby increasing our confidence in these results. It is noteworthy that all compounds were accommodated by the simulations at the catalytic site, some of them with considerable energy prediction. While the energy predictions are weaker than NDGA, it should be noted that, given the number of compounds, they may cooperate and exhibit competitive inhibition. Fascinatingly, the compound longifolene binds in the vicinity of Leu414. This amino acid residue is important for substrate positioning during the catalytic event, along with Leu368, Leu607, and Ile406 [34], which were also predicted to interact with the compounds. Arg596 appeared frequently in molecular docking simulations and is one of the interaction sites with NDGA. His372 also appears several times in the interactions. It is one of the amino acid residues that coordinate the catalytic iron, so it is possible that the compounds block access to the iron, as with NDGA [16].

It is noteworthy that the compounds studied here are quite small, so there is a low likelihood that they can access the catalytic site without the conformational changes required to accommodate larger substrates or inhibitors. It has been established that 5LO inhibition is experimentally linked to the activation of apoptosis pathways. Hence, predictions point to a mechanism of action involving this enzyme and explain the effects observed in Huh-7 hepatoma cells. The cytotoxic potency of AKBA (IC_50_ around 5 μM) compares favorably with multiple established anti-hepatocarcinoma agents, such as Sorafenib [35] and Doxorubicin [36]. Critically, the cytotoxicity of AKBA is mediated by caspase-dependent apoptosis, a mechanism distinct from kinase inhibition (sorafenib) or DNA alkylation/topoisomerase inhibition (doxorubicin), representing a non-overlapping mechanism of resistance that potentially enables synergistic combination approaches [36].

The hepatoprotective properties documented for AKBA and other boswellic acids in preclinical models are especially remarkable [10,11,12], as they contrast with the significant hepatotoxicity associated with conventional chemotherapy. This suggests potential for a superior therapeutic window and reduced off-target organ toxicity, motivating further preclinical investigation in hepatotoxicity assays and animal models. The limitations of this study include support on a single hepatocellular carcinoma cell line (Huh-7) and the lack of direct assessment of 5LO activation. In future research, the incorporation of additional cell lines, extended treatment durations, serial dosing studies, enzymatic kinetics, and in vivo models should be investigated.

## 4. Materials and Methods

### 4.1. Plant Material and Essential Oil Extraction

*Boswellia serrata* resin (8 kg) was procured from 99exports herbs (http://99exports.com/, accessed on 30 June 2026). Essential oil was isolated by steam distillation at 100 °C (2 h) from 100 g of resin, using a Clevenger-type apparatus. The resulting distillate was subjected to liquid–liquid partitioning with chloroform to remove polar impurities, and the mixture was concentrated under reduced pressure with a rotary evaporator (Büchi R-210, Büchi Labortechnik AG, Flawil, Switzerland) at 40 °C. The concentrated essential oil was stored at −20 °C in amber glass bottles under a nitrogen atmosphere until use.

### 4.2. Phytochemical Characterization

The essential oil was analyzed by GC-MS using an Agilent 7890A gas chromatograph coupled to an Agilent 5975C quadrupole mass spectrometer (Agilent Technologies, Waldbronn, Germany). Analysis used an HP-5MS capillary column (30 m × 0.25 mm ID, 0.25 μm film thickness) with helium as carrier gas at 1.0 mL/min. Oven temperature was programmed from 60 °C (1 min hold) to 280 °C (10 min hold) at 5 °C/min. The MS was operated in electron ionization mode (70 eV) with a scan range of *m*/*z* 40–550. Individual components were identified by comparing retention times and mass spectral fragmentation patterns with the NIST 14 mass spectral database and published literature values [37].

### 4.3. AKBA Reagent

Acetyl-11-keto-β-boswellic acid (AKBA; CAS 67416-61-9; ≥98% HPLC purity) was purchased from Cayman Chemical Company (Ann Arbor, MI, USA) and was stored at 4 °C protected from light.

### 4.4. Cell Culture

Human hepatocellular carcinoma-derived Huh-7 cells were obtained from the Japanese Cancer Research Resources Bank (JCRB) and maintained in Williams’ medium (Gibco/Thermo Fisher Scientific, Waltham, MA, USA) supplemented with 10% fetal bovine serum (FBS), 100 U/mL penicillin, and 100 μg/mL streptomycin. Cells were cultured in a 37 °C incubator with a 5% CO_2_ atmosphere and sub-cultured every 3–4 days using 0.25% trypsin-EDTA (Gibco/Thermo Fisher Scientific, Waltham, MA, USA) upon reaching 80–90% confluency. Cell viability was confirmed by trypan blue exclusion (≥95%) before seeding for experiments.

### 4.5. Experimental Treatments and Preliminary Dose–Response Studies

To determine optimal concentrations, preliminary dose–response experiments were conducted. The essential oil was dissolved in dimethyl sulfoxide (DMSO; Sigma-Aldrich, St. Louis, MO, USA) to prepare stock solutions, which were then diluted to achieve final concentrations of 1, 2, 4, and 8 μg/mL in culture medium. AKBA was diluted to achieve final concentrations of 5, 10, 20, 40, and 80 μM. These concentrations were informed by prior literature [10,11,12]. Vehicle control was DMSO concentrations (0.05–0.1% final concentration) without active compound. Untreated control wells received only culture medium.

### 4.6. Cytotoxicity Assay

Cell proliferation and viability were evaluated using the Cell Counting Kit-8 (CCK-8; Dojindo Laboratories, Kumamoto, Japan) [38]. Huh-7 cells were seeded in 96-well flat-bottom tissue culture plates at a density of 5 × 10^3^ cells/well in 100 μL of culture medium. After 24 h, cells were treated with vehicle (control), various concentrations of essential oil or AKBA, or left untreated (*n* = 4 replicates per condition). At 24, 48, and 72 h post-treatment, 10 μL of CCK-8 solution was added to each well, the plates were incubated for 2 h at 37 °C, and absorbance was measured at 450 nm using a microplate reader (Biotek Synergy 2, Winooski, VT, USA). Absorbance (culture medium alone) was subtracted from all measurements. Cell viability was expressed as a percentage of untreated controls, with viability (%) = (absorbance of treated cells/absorbance of control cells) × 100. IC_50_ values were determined using GraphPad Prism version 8.0.

### 4.7. Three-Dimensional Spheroid Culture

Huh-7 cells were seeded in ultra-low attachment 96-well plates (Corning, Falcon, NY, USA) at 5 × 10^3^ cells/well in Williams’ medium supplemented with 10% FBS and incubated at 37 °C in 5% CO_2_ for 5 days to permit spheroid formation. Mature spheroids (typically 100–200 μm in diameter) were treated with vehicle control (DMSO 0.05%) or selected concentrations of essential oil (1 μg/mL) and AKBA (1 μM) based on monolayer dose–response results (IC_50_ or sub-IC50 concentrations). Spheroid cultures were maintained for an additional 72 h post-treatment. At harvest, spheroids were manually counted via light microscopy using a Nikon Eclipse inverted microscope (Tokyo, Japan) at 4× magnification, with spheroid number and diameter (calculated as the mean of the perpendicular diameters) recorded for each well. Data are presented as fold-change in spheroid number and mean diameter relative to untreated controls.

### 4.8. Protein Extraction

Huh-7 cells were seeded at 2 × 10^5^ cells/well in 6-well plates in Williams’ medium supplemented with 10% FBS. After 24 h, cells were treated with vehicle control (DMSO 0.05%), essential oil (1 μg/mL), or AKBA (sub-IC50 1 μM) for 72 h. At harvest, culture medium was aspirated, cells were washed twice with ice-cold phosphate-buffered saline (PBS; pH 7.4), and were lysed in 500 μL of modified RIPA lysis buffer (Millipore-Sigma, Darmstadt, Germany) supplemented with protease inhibitor cocktail (Roche Diagnostics, Basel, Switzerland) and phosphatase inhibitors (1 mM sodium orthovanadate, 1 mM sodium fluoride) for 15 min on ice. Cell lysates were centrifuged at 13,000× *g* for 10 min at 4 °C, and the supernatant (total protein extract) was transferred to sterile microtubes and stored at −80 °C until analysis. Total protein concentration was quantified using the bicinchoninic acid (BCA) assay (Pierce Chemical, Rockford, IL, USA) following the manufacturer’s protocol. Briefly, 10 μL of protein extract was incubated with BCA reagent for 30 min at 37 °C, and absorbance was measured at 562 nm. Protein concentration was determined by comparison with a standard curve of bovine serum albumin (BSA) (0–2 mg/mL).

### 4.9. Protein Expression Analysis by Western Blot

Protein extracts (30 μg per lane) were denatured by heating at 95 °C for 5 min in Laemmli sample buffer (62.5 mM Tris-HCl, pH 6.8, 25% glycerol, 2% SDS, 5% β-mercaptoethanol, 0.01% bromophenol blue). They were separated by sodium dodecyl sulfate-polyacrylamide gel electrophoresis (SDS-PAGE) on 10–12% gradient acrylamide gels at 120 V for 90 min in running buffer (25 mM Tris, 192 mM glycine, 0.1% SDS; pH 8.3). Proteins were then transferred to PVDF membranes (Millipore-Sigma) by electroblotting (120 V, 120 min, 4 °C) in transfer buffer (25 mM Tris, 192 mM glycine, 20% methanol; pH 8.3). Membranes were blocked with Tris-buffered saline containing 0.1% Tween-20 (TBS-T) and 5% non-fat milk for 1 h at room temperature under gentle agitation.

Primary antibodies were diluted in blocking solution and incubated with membranes overnight at 4 °C under constant agitation. The following primary antibodies were employed: anti-cyclin D1 (Santa Cruz Biotechnology, Dallas, TX, USA. SC-753, rabbit, 1:500), anti-CDK4 (Santa Cruz, TX, USA. SC-749, mouse, 1:500), anti-p21 (WAF1/CIP1) (Santa Cruz, TX, USA. SC-756, mouse, 1:500), anti-E-cadherin (Santa Cruz, TX, USA. SC-21791, mouse, 1:500), anti-EpCAM (Cell Signaling Technology, Danvers, MA, USA. 2929, rabbit, 1:1000), anti-AFP (Cell Signaling Technology, MA, USA. 3903, rabbit, 1:1000), anti-Mcl-1 (Abcam, Cambridge, UK. ab5714, mouse, 1:1000), anti-cleaved caspase-3 (Cell Signaling Technology, MA, USA. 9661, rabbit, 1:1000), and anti-β-actin (Santa Cruz, TX, USA. SC-47778, mouse, 1:5000) as the loading control.

Membranes were then washed three times (10 min each) in TBS-T and incubated with appropriate HRP-conjugated secondary antibodies (Santa Cruz Biotechnology; anti-mouse or anti-rabbit at 1:5000) for 1 h at room temperature. After three additional washes in TBS-T, chemiluminescent detection was performed using an enhanced chemiluminescence (ECL) substrate (Pierce SuperSignal West Pico Chemiluminescent Substrate; Thermo Fisher Scientific), and images were acquired on a ChemiDoc Imaging System (Bio-Rad Laboratories, Hercules, CA, USA). Band densities were quantified using ImageJ (version 1.52a; NIH, Bethesda, MD, USA), with values normalized to the β-actin loading control and expressed as fold change relative to untreated controls.

### 4.10. In Silico Analysis

Ligand preparation. The compounds were downloaded from the PubChem database (Table 1). Their protonation states were established at pH 7.4, and geometries were optimized using the MMFF94 force field in OpenBabel (Version 3.1.1) [39]. They were converted to PDBQT format using the Python (Version (3.11.0) script prepare_ligand4.py included in AutoDockTools/MGLTools (Version 1.5.7) [40]. They were then visually inspected using the PyMOL Molecular Graphics System (Version 3.1; Schrödinger, LLC, New York, NY, USA).

The crystallographic structures of the 5LO protein were downloaded from the Protein Data Bank (PDB) database (Table 3). Unnecessary structures were removed: bound ligands, water molecules (none were mediating ligand-protein interactions), and twin proteins included in the structure because they did not contain co-crystallized ligands. Hydrogens were added using UCSF Chimera (Version 1.19) [41]. For 6N2W, gaps in the crystallographic structure were simulated with MODELLER (Version 10.6) [42]. Nonpolar hydrogens were merged using AutoDockTools/MGLTools, Gasteiger charges were calculated, and the proteins were converted to PDBQT format. Finally, they were visually inspected to rule out structural aberrations.

For the molecular docking, the procedure for calculating the center and size of the simulation boxes (Table 4) was as follows: Using Python scripts, for each atom of the co-crystallized ligand, amino acid (AA) residues were identified at a maximum distance of 5 Angstroms. The maximum and minimum coordinates on each axis of the component atoms of the AAs were identified to conceptualize a cube, from which the center and size were calculated. For all simulations, 200 docked poses were generated for each experimental ligand: 100 poses obtained with the AD4 force field of AutoDock-GPU (Version 1.6) [43] and another 100 with the Vina force field of Vina-GPU (Version 2.1) [44]. Subsequently, energy score calculations (for poses obtained with the AD4 force field) were standardized using the Vina force field with the AutoDock Vina program (Version 1.2.7) [45] in “score only” mode. The compounds in Table 1 include AKBA and NDGA for redocking analysis; for this purpose, the root-mean-square deviation (RMSD) was calculated using the DockRMSD program (Version 1.1) [46]. The simulations were run on a system with Ubuntu 25.10, an AMD Ryzen 5 7600X processor, an AMD Radeon RX 6600 graphics card, and 32 GB of RAM.

### 4.11. Statistical Analysis

Data from a minimum of three independent experiments (*n* = 3–4 replicates per condition per experiment) were analyzed using one-way or two-way analysis of variance (ANOVA) with post hoc tests (Dunnett’s or Tukey’s multiple comparison test) via GraphPad Prism 8.0 software. Significance was accepted at *p* < 0.05 with 95% confidence intervals. Data are presented as mean ± standard error of the mean (SEM). IC_50_ values were determined by non-linear regression using a four-parameter logistic equation.

## 5. Conclusions

The results contribute to an understanding of the mechanistic basis of the traditional use of *B. serrata* resin in medicinal systems and provide preclinical support for the further development of its essential oil and boswellic acid-derived therapeutics for hepatocellular carcinoma. This investigation provides comprehensive in vitro and in silico evidence that *B. serrata* essential oil and purified AKBA exert potent cytotoxic effects on Huh-7 hepatocellular carcinoma cells by activating caspase-3-mediated apoptosis via 5LO inhibition.

Cytotoxic effects are preserved in more physiologically relevant three-dimensional spheroid cultures, implying possible translational significance. The mechanistic profile—involving caspase-dependent apoptosis in the absence of substantial cell-cycle checkpoint disruption or epithelial–mesenchymal transition—indicates a distinct mechanism of action from conventional chemotherapy and targeted kinase inhibitors, with consequences for overcoming acquired resistance. In silico analysis predicted that the compounds in B. serrata essential oil are competitive inhibitors of the 5LO enzyme, which activates pro-apoptotic pathways in cancer cells.

These data support further investigation of AKBA and the main components of the essential oil as lead compounds for preclinical development in animal models of hepatocellular carcinoma, including evaluation of in vivo efficacy, pharmacokinetics, toxicity, and combination approaches with contemporary therapeutics. Multi-center clinical trials evaluating *B. serrata* derivatives in HCC patients, potentially in combination with checkpoint immunotherapy or kinase inhibitors, are justified by the accumulated preclinical evidence and represent a hopeful pathway to expand the therapeutic options available to HCC patients.

## Figures and Tables

**Table 1:** Essential oil compounds that were studied in the molecular docking simulations.

Name	Synonym	PubChem Compund Identifier	RT	Area (%)
2-Ethyl-4,5-dimethylphenol	3,4-dimethyl-6-ethylphenol	247477	8.287	0.66
2-Hydroxy-5-methylacetophenone	O-acetyl-p-cresol	15068	8.287	0.66
Beta-Thujene	2-thujene	520384	5.000	0.82
Chrysanthenone	2-pinen-7-one	442463	7.622	1.49
Cis-Verbanone	-	12304644	7.622	1.49
Durenol	2,3,5,6-tetramethylphenol	10694	8.287	0.66
Elemicin	3,4,5-trimethoxyallylbenzene	10248	9.922	0.53
Isoterpinolene	P-mentha-2,4(8)-diene	102443	7.461	2.96
Linalool	Linalyl alcohol	6549	6.531	1.55
Longifolene	Junipene	1796220	9.233	0.82
Methyleugenol	Eugenol methyl ether	7127	8.968	1.53
p-Cymen-8-ol	Dimethyl-p-tolyl carbinol	14529	7.389	2.00
P-Cymene	Dolcymene	7463	5.882	1.42
Terpinen-4-ol	4-carvomenthenol	11230	7.357	7.81
Terpinolene	Isoterpinene	11463	7.461	2.96
Valencene	(3r,4as,5r)-4a,5-dimethyl-3-(prop-1-en-2-yl)-1,2,3,4,4a,5,6,7-octahydronaphthalene	9855795	9.233	0.82
α-Terpineol	Alpha-terpineol	17100	7.461	2.96
Acetyl-11-keto-β-boswellic acid *	AKBA	11168203	-	-
Dimethyl sulfoxide *	DMSO	679	-	-
Nor-dihydro-guaretic acid *	NDGA	4534	-	-

* Reference molecules for the docking studies. RT = Retention time.

**Table 2:** Energy forecasts from molecular docking simulations.

Compound	Binding Energy (kcal/mol)	PubChem Identifier
NDGA	−8.3	4534
Longifolene	−6.7	1796220
Valencene	−6.5	9855795
2-Ethyl-4,5-dimethylphenol	−6.5	247477
Terpinolene	−6.4	11463
p-Cymen-8-ol	−6.3	14529
Isoterpinolene	−6.2	102443
α-Terpineol	−6.2	17100
Durenol	−6.2	10694
2-Hydroxy-5-methylacetophenone	−6	15068
P-Cymene	−6	7463
Elemicin	−5.9	10248
Terpinen-4-ol	−5.8	11230
Chrysanthenone	−5.7	442463
β-Thujene	−5.7	520384
Methyleugenol	−5.6	7127
Cis-Verbanone	−5.6	12304644
Linalool	−5.4	6549
DMSO	−2.7	679

**Table 3:** Parameters of the chosen crystallographic structures.

Protein	PDB ID	Organism	Mutations	Resolution (Å)	Bound Ligand	Deposited
5LO	6N2W	*H. sapiens*	-	2.71	NDGA	14 November 2018
5LO	6NCF	*H. sapiens*	amino-terminal domain	2.87	AKBA	11 December 2018

**Table 4:** Configuration of the molecular docking simulation boxes. All units are given in Angstroms (Å).

PDB ID	Site	x Center	y Center	z Center	x Size	y Size	z Size
6N2W	Catalytic site	36.067	64.433	37.706	17.625	21.75	26.625
6NCF	AKBA binding site	15.337	−20.793	−18.091	24.75	17.25	21

## Data Availability

The original contributions presented in this study are included in the article/Supplementary Materials. Further inquiries can be directed to the corresponding author.

## Supplementary Materials

The following supporting information can be downloaded at: https://www.mdpi.com/article/10.3390/ijms27135978/s1.

## Abbreviations

The following abbreviations are used in this manuscript:

AKBA	acetyl-11-keto-β-boswellic acid
5LO	5-lipoxygenase
HCC	Hepatocellular carcinoma
EpCAM	epithelial cell adhesion molecule
AFP	alpha-fetoprotein
ECL	enhanced chemiluminescence
FLAP	5LO activator protein
