# Supplementary material for: Cytotoxic Activity of Boswellia serrata Roxb. Essential Oil and Acetyl-11-Keto-β-Boswellic Acid (AKBA) on Hepatocellular Carcinoma Cells: In Vitro and In Silico Study
Source: Int J Mol Sci. 2026 Jul 3;27(13):5978. doi: 10.3390/ijms27135978 (PMC13362177; doi:10.3390/ijms27135978)
Supplement: Supplementary file 1 [file ijms-27-05978-s001.zip › ijms-4367394-supplementary.pdf]

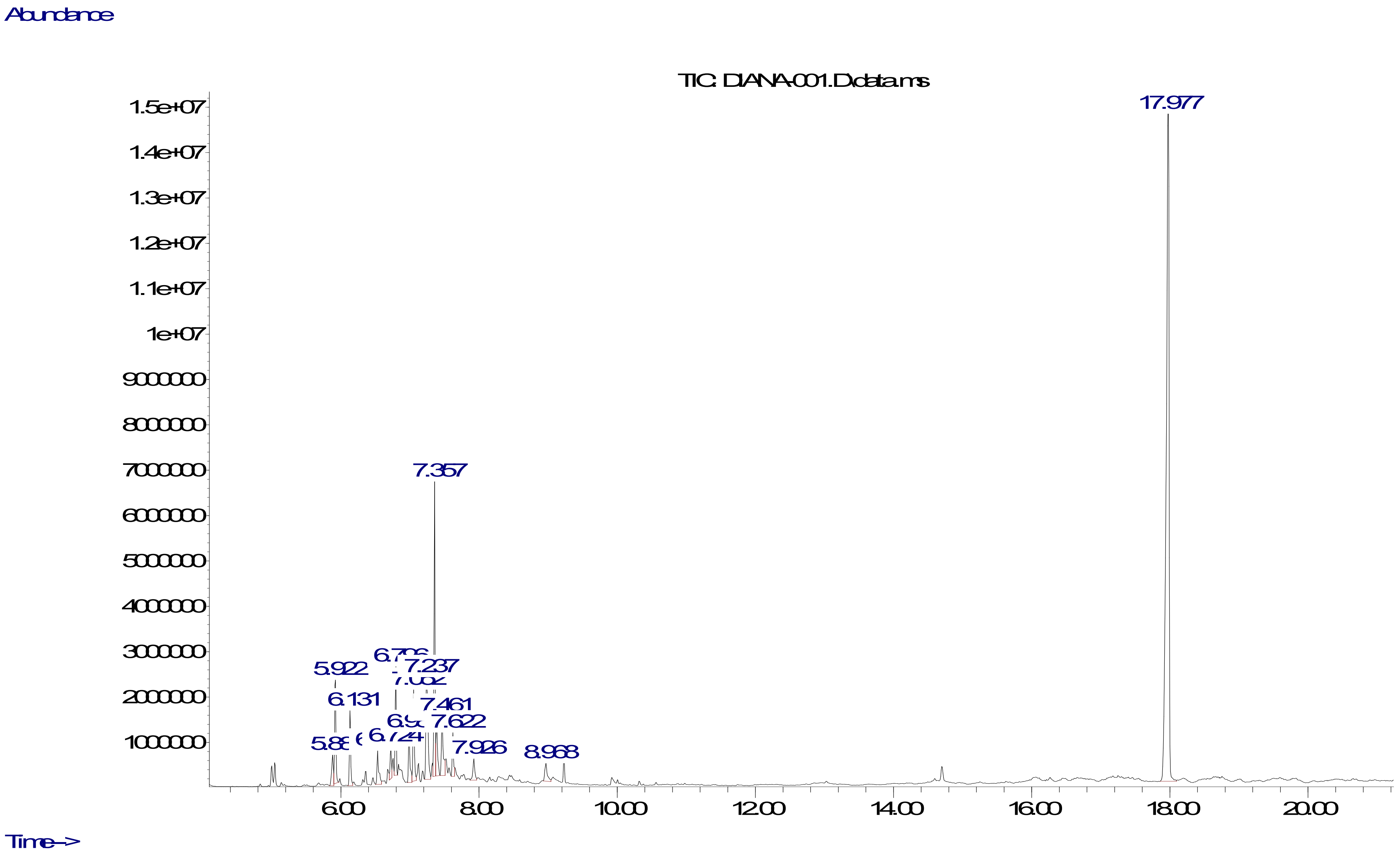
Abundance
TIC: DIANA-001.D\data.ms
17.977
7.357
5.922
6.131
5.88
6.724
6.796
7.237
7.052
7.461
6.9
7.622
7.926
8.968
Time-->

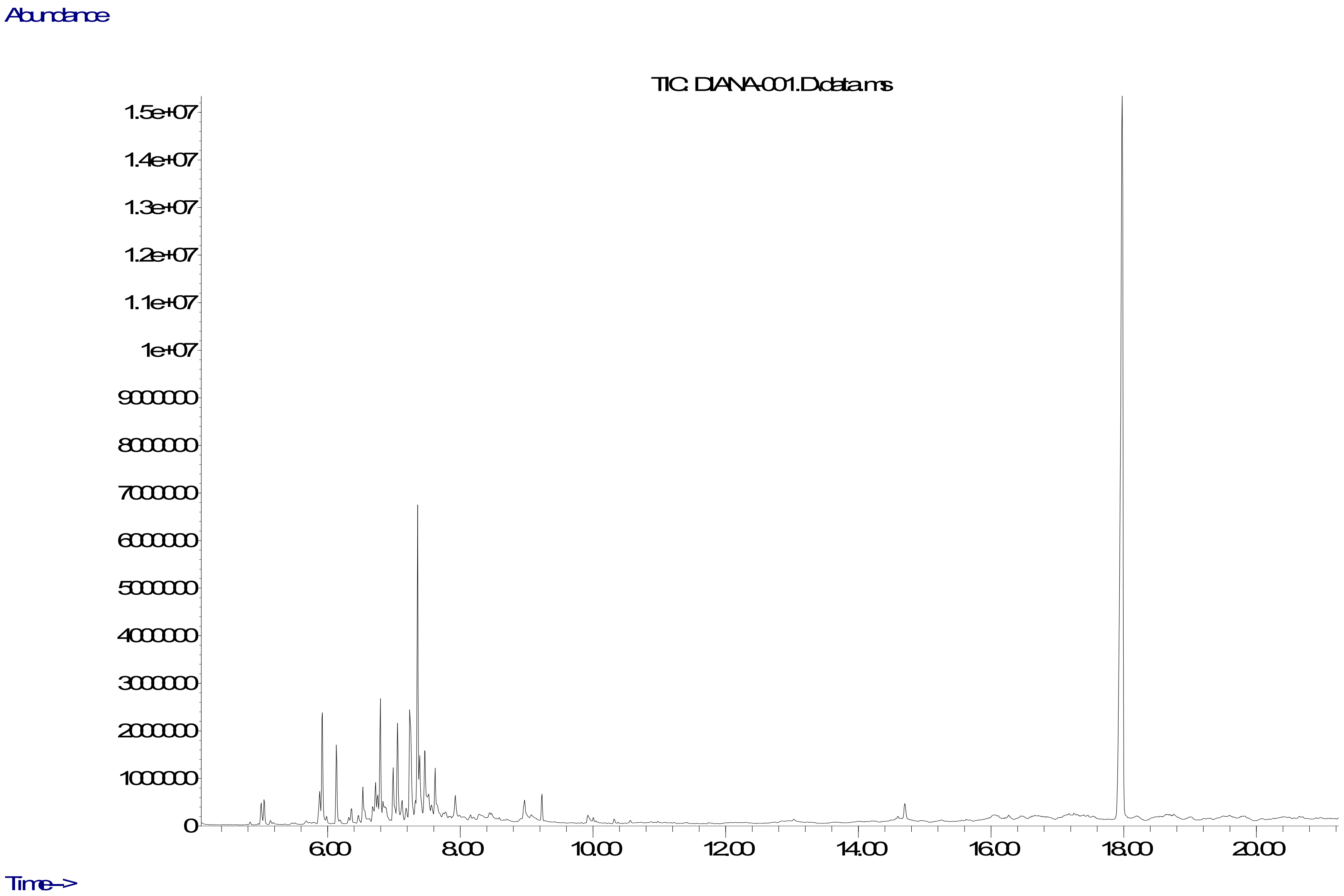

Abundance
TIC: DIANA-001.D\data.ms
1.5e+07
1.4e+07
1.3e+07
1.2e+07
1.1e+07
1e+07
9000000
8000000
7000000
6000000
5000000
4000000
3000000
2000000
1000000
0
6.00
8.00
10.00
12.00
14.00
16.00
18.00
20.00
Time-->

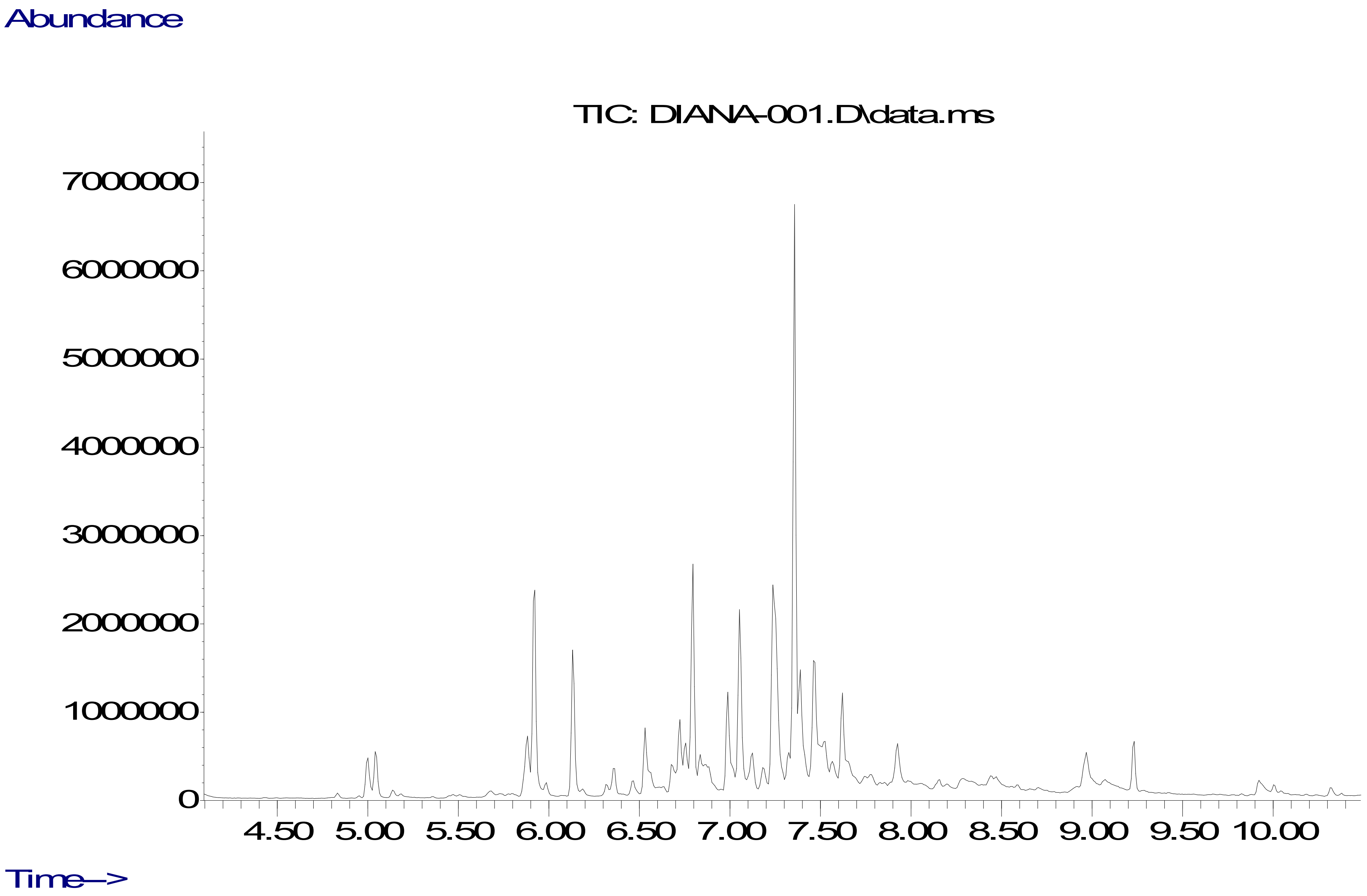

Abundance
TIC: DIANA-001.D\data.ms
7000000
6000000
5000000
4000000
3000000
2000000
1000000
0
4.50
5.00
5.50
6.00
6.50
7.00
7.50
8.00
8.50
9.00
9.50
10.00
Time-->

Library Search Report

Data Path : D:\1\DATA\MARGARITA\
Data File : DIANA-001.D
Acq On : 23 Nov 2017 2:46
Operator :
Sample : ACEITE ESCENCIAL B. S.
Misc : 3 mg en 1 mL
ALS Vial : 1 Sample Multiplier: 1

Search Libraries: C:\Database\NIST08.L Minimum Quality: 0

Unknown Spectrum: Apex
Integration Events: RTE Integrator - rteint2desiempre.p

| Pk# | RT | Area% | Library/ID | Ref# | CAS# | Qual |
|---|---|---|---|---|---|---|
| 1 | 5.000 | 0.82 | C:\Database\NIST08.L | | | |
| | | | Bicyclo[3.1.0]hex-2-ene, 4-methylene-1-(1-methylethyl)- | 14769 | 036262-09-6 | 86 |
| | | | Benzene, (2-methylpropyl)- | 14672 | 000538-93-2 | 64 |
| | | | Spiro[2.4]hepta-4,6-diene | 2453 | 000765-46-8 | 62 |
| 2 | 5.041 | 0.82 | C:\Database\NIST08.L | | | |
| | | | Bicyclo[3.1.0]hex-2-ene, 4-methylene-1-(1-methylethyl)- | 14769 | 036262-09-6 | 86 |
| | | | Benzene, butyl- | 14647 | 000104-51-8 | 64 |
| | | | Cyclobutene, 2-propenylidene- | 2456 | 052097-85-5 | 64 |
| 3 | 5.882 | 1.42 | C:\Database\NIST08.L | | | |
| | | | Benzene, 1-methyl-4-(1-methylethyl)- | 14731 | 000099-87-6 | 97 |
| | | | Benzene, 1-methyl-2-(1-methylethyl)- | 14737 | 000527-84-4 | 94 |
| | | | Benzene, 1-methyl-3-(1-methylethyl)- | 14732 | 000535-77-3 | 94 |
| 4 | 5.922 | 3.76 | C:\Database\NIST08.L | | | |
| | | | Ethanone, 1-(1,4-dimethyl-3-cyclohexen-1-yl)- | 24829 | 043219-68-7 | 50 |
| | | | trans-3(10)-Caren-2-ol | 24683 | 1000151-66-5 | 50 |
| | | | 3-Formyl-1-methyl-2(1H)-pyridone | 16479 | 1000306-46-7 | 38 |
| 5 | 6.131 | 2.46 | C:\Database\NIST08.L | | | |
| | | | trans-3(10)-Caren-2-ol | 24683 | 1000151-66-5 | 53 |
| | | | Ethanone, 1-(1,4-dimethyl-3-cyclohexen-1-yl)- | 24829 | 043219-68-7 | 53 |
| | | | Ethanone, 1-(1,4-dimethyl-3-cyclohexen-1-yl)- | 24827 | 043219-68-7 | 53 |
| 6 | 6.355 | 0.56 | C:\Database\NIST08.L | | | |
| | | | Tricyclo[2.2.1.0(2,6)]heptane | 2601 | 000279-19-6 | 60 |
| | | | 1-Methylcyclohexa-1,3-diene | 2595 | 1000298-96-0 | 55 |
| | | | 3-Methyl-4-cyclohexene-1,2-dicarbo | 35022 | 005333-84-6 | 53 |

xylic anhydride

7 6.531 1.55 C:\Database\NIST08.L
1,6-Octadien-3-ol, 3,7-dimethyl- 26281 000078-70-6 93
1,6-Octadien-3-ol, 3,7-dimethyl- 26287 000078-70-6 70
1,6-Octadien-3-ol, 3,7-dimethyl- 26288 000078-70-6 53

8 6.676 0.65 C:\Database\NIST08.L
trans-.alpha.-Bergamotene 62324 013474-59-4 30
Camphene 15490 000079-92-5 25
Camphene 15489 000079-92-5 20

9 6.724 1.08 C:\Database\NIST08.L
2-(5-Methyl-furan-2-yl)-propionald 17166 1000193-72-3 50
ehyde
3-Octyn-2-one 10361 001119-58-0 47
Pyridine, 3-methoxy- 5508 007295-76-3 43

10 6.756 0.59 C:\Database\NIST08.L
Ethanone, 1-(1,4-dimethyl-3-cycloh 24827 043219-68-7 59
exen-1-yl)-
(1,3-Dimethyl-2-methylene-cyclopen 18562 1000190-16-2 50
tyl)-methanol
2,3-Dehydro-1,8-cineole 24688 092760-25-3 50

11 6.796 3.11 C:\Database\NIST08.L
Thujone 24635 000546-80-5 87
Methyl ethyl cyclopentene 5909 019780-56-4 76
Thujone 24636 000546-80-5 74

12 6.988 2.02 C:\Database\NIST08.L
Bicyclo[3.1.0]hexan-3-ol, 4-methyl 24951 003310-02-9 59
ene-1-(1-methylethyl)-, (1.alpha.,
3.alpha.,5.alpha.)-
Tricyclo[3.2.1.0(2,4)]octane, 3-me 9353 1000150-04-5 58
thylene-
1-Cyclopentene, 1-(methylenecyclop 9352 1000153-40-5 58
ropyl)-

13 7.052 3.09 C:\Database\NIST08.L
3-Decyne 16633 002384-85-2 52
1-(1-Butyny)cyclopentanol 17318 1000342-86-5 50
Pyridine, 4-methyl-, 1-oxide 5533 001003-67-4 38

14 7.125 0.80 C:\Database\NIST08.L
Methyl ethyl cyclopentene 5909 019780-56-4 49
1-Ethyl-5-methylcyclopentene 5934 097797-57-4 49
3-Heptyne, 5-methyl- 5870 061228-09-9 49

15 7.181 0.51 C:\Database\NIST08.L
5-Eicosyne 119876 074685-31-7 47
Cyclopentane, 1-isobutylidene-3-me 16758 1000150-62-1 30
thyl-
Cyclopropane, 1-methyl-1-isopropen 2905 003422-07-9 27
yl-

16 7.237 5.74 C:\Database\NIST08.L
Tricyclo[3.2.1.0(2,4)]octane, 3-me 9353 1000150-04-5 58
thylene-
Bicyclo[3.1.0]hex-2-ene, 4-methyle 14769 036262-09-6 38
ne-1-(1-methylethyl)-
2,4-Methano-1H-cycloprop[cd]indene 14758 1000337-06-4 35
, octahydro-

17 7.357 7.81 C:\Database\NIST08.L
3-Cyclohexen-1-ol, 4-methyl-1-(1-m 26431 020126-76-5 94
ethylethyl)-, (R)-
3-Cyclohexen-1-ol, 4-methyl-1-(1-m 26398 000562-74-3 94
ethylethyl)-
3-Cyclohexen-1-ol, 4-methyl-1-(1-m 26428 020126-76-5 87
ethylethyl)-, (R)-

18 7.389 2.00 C:\Database\NIST08.L
Benzenemethanol, .alpha.,.alpha.,4 23473 001197-01-9 87
-trimethyl-
Phenol, 2-ethyl-4,5-dimethyl- 23367 002219-78-5 68
Silane, trimethylphenyl- 24219 000768-32-1 64

19 7.461 2.96 C:\Database\NIST08.L
3-Cyclohexene-1-methanol, .alpha., 26490 010482-56-1 94
.alpha.,4-trimethyl-, (S)-
Cyclohexene, 3-methyl-6-(1-methyle 15666 000586-63-0 91
thylidene)-
Cyclohexene, 1-methyl-4-(1-methyle 15665 000586-62-9 83
thylidene)-

20 7.525 0.51 C:\Database\NIST08.L
1,6-Dimethylhepta-1,3,5-triene 9929 1000196-61-0 46
3-Caren-10-al 23292 1000151-86-0 43
3,5-Heptadienal, 2-ethylidene-6-me 23430 099172-18-6 43
thyl-

21 7.622 1.49 C:\Database\NIST08.L
Bicyclo[3.1.1]hept-3-en-2-one, 4,6 23510 001196-01-6 95
,6-trimethyl-, (1S)-
Bicyclo[3.1.1]hept-3-en-2-one, 4,6 23508 001196-01-6 93
,6-trimethyl-, (1S)-
Bicyclo[3.1.1]hept-2-en-6-one, 2,7 23482 000473-06-3 90
,7-trimethyl-

22 7.926 1.06 C:\Database\NIST08.L
1-Cyclohexene-1-carboxaldehyde, 4- 24889 021391-98-0 62
(1-methylethyl)-
2,4-Hexadienal, (E,E)- 2785 000142-83-6 60
2,4-Hexadienal, (E,E)- 2784 000142-83-6 49

23 8.287 0.66 C:\Database\NIST08.L
Phenol, 2,3,5,6-tetramethyl- 23359 000527-35-5 90
Ethanone, 1-(2-hydroxy-5-methylphe 24133 001450-72-2 86
nyl)-
Phenol, 2-ethyl-4,5-dimethyl- 23367 002219-78-5 83

24   8.968  1.53 C:\Database\NIST08.L
Benzene, 1,2-dimethoxy-4-(2-propen  42825 000093-15-2 96
yl)-
Benzene, 1,2-dimethoxy-4-(2-propen  42826 000093-15-2 93
yl)-
Benzene, 1,2-dimethoxy-4-(2-propen  42824 000093-15-2 78
yl)-

25   9.072  0.81 C:\Database\NIST08.L
Phenol, 4-(trifluoromethoxy)-       43324 000828-27-3 38
Phosphonothioic difluoride, phenyl  43242 000657-40-9 35
Phenyl trifluoromethyl sulfide      43329 000456-56-4 30

26   9.233  0.82 C:\Database\NIST08.L
1,4-Methanoazulene, decahydro-4,8,  62484 000475-20-7 99
8-trimethyl-9-methylene-, [1S-(1.a
lpha.,3a.beta.,4.alpha.,8a.beta.)]
1,4-Methanoazulene, decahydro-4,8,  62478 000475-20-7 98
8-trimethyl-9-methylene-, [1S-(1.a
lpha.,3a.beta.,4.alpha.,8a.beta.)]
Naphthalene, 1,2,3,5,6,7,8,8a-octa  62508 004630-07-3 96
hydro-1,8a-dimethyl-7-(1-methyleth
enyl)-, [1R-(1.alpha.,7.beta.,8a.a
lpha.)]-

27   9.922  0.53 C:\Database\NIST08.L
Benzene, 1,2,3-trimethoxy-5-(2-pro  65315 000487-11-6 94
penyl)-
Benzene, 1,2,3-trimethoxy-5-(2-pro  65316 000487-11-6 94
penyl)-
Benzene, 1,2,3-trimethoxy-5-(2-pro  65317 000487-11-6 90
penyl)-

28  14.699  0.97 C:\Database\NIST08.L
Cyclopropaneoctanoic acid, 2-[[2-[ 161870 010152-71-3 43
(2-ethylcyclopropyl)methyl]cyclopr
opyl]methyl]-, methyl ester
Cyclohexanol, 5-methyl-2-(1-methyl  26502 000089-79-2 38
ethenyl)-, [1R-(1.alpha.,2.beta.,5
.alpha.)]-
Cyclohexanol, 5-methyl-2-(1-methyl  26504 000089-79-2 38
ethenyl)-, [1R-(1.alpha.,2.beta.,5
.alpha.)]-

29  17.977 49.32 C:\Database\NIST08.L
1,2-Benzenedicarboxylic acid, mono 119596 004376-20-9 91
(2-ethylhexyl) ester
1,2-Benzenedicarboxylic acid, diis 192055 027554-26-3 91
ooctyl ester
Phthalic acid, neopentyl pentyl es 141210 1000315-29-8 68
ter

30  18.755  0.54 C:\Database\NIST08.L
Dotriacontyl trifluoroacetate      216734 1000351-75-4 55
Hexatriacontyl trifluoroacetate    218298 1000351-88-6 55
Tetradecane, 1-bromo-              118049 000112-71-0 53

TOXINAT2.M Thu Nov 23 03:31:02 2017

Area Percent Report

Data Path : D:\1\DATA\MARGARITA\
Data File : DIANA-001.D
Acq On : 23 Nov 2017 2:46
Operator :
Sample : ACEITE ESCENCIAL B. S.
Misc : 3 mg en 1 mL
ALS Vial : 1 Sample Multiplier: 1

Integration Parameters: rteint2desiempre.p
Integrator: RTE
Smoothing : OFF Filtering: 5
Sampling : 1 Min Area: 1 % of largest Peak
Start Thrs: 0.1 Max Peaks: 100
Stop Thrs : 0.05 Peak Location: TOP

If leading or trailing edge < 100 prefer < Baseline drop else tangent >
Peak separation: 5

Method : C:\msdchem\1\DATA\2010\04\16\Run-2010-04-16-001403593UTC\TOXINAT2.M
Title :

Signal : TIC: DIANA-001.D\data.ms

| peak # | R.T. min | first scan | max scan | last scan | PK TY | peak height | corr. area | corr. % max. | % of total |
|---|---|---|---|---|---|---|---|---|---|
| 1 | 5.001 | 110 | 114 | 117 | rBV | 451733 | 708895 | 1.66% | 0.817% |
| 2 | 5.041 | 117 | 119 | 127 | rVB | 521702 | 708961 | 1.66% | 0.817% |
| 3 | 5.882 | 218 | 224 | 226 | rBV | 683180 | 1234879 | 2.89% | 1.424% |
| 4 | 5.922 | 226 | 229 | 235 | rVV | 2335851 | 3258531 | 7.62% | 3.757% |
| 5 | 6.131 | 251 | 255 | 260 | rBV | 1655512 | 2129524 | 4.98% | 2.455% |
| 6 | 6.355 | 280 | 283 | 293 | rVB | 302155 | 489460 | 1.14% | 0.564% |
| 7 | 6.531 | 301 | 305 | 312 | rBV2 | 746676 | 1342459 | 3.14% | 1.548% |
| 8 | 6.676 | 320 | 323 | 326 | rBV3 | 310734 | 564074 | 1.32% | 0.650% |
| 9 | 6.724 | 326 | 329 | 331 | rVV | 727881 | 939394 | 2.20% | 1.083% |
| 10 | 6.756 | 331 | 333 | 335 | rVV | 400453 | 510313 | 1.19% | 0.588% |
| 11 | 6.796 | 335 | 338 | 341 | rVB | 2398539 | 2694521 | 6.30% | 3.106% |
| 12 | 6.988 | 359 | 362 | 367 | rBV | 1109440 | 1748999 | 4.09% | 2.016% |
| 13 | 7.052 | 367 | 370 | 375 | rVV | 2009966 | 2679142 | 6.26% | 3.089% |
| 14 | 7.125 | 375 | 379 | 383 | rVB3 | 408621 | 695169 | 1.62% | 0.801% |
| 15 | 7.181 | 383 | 386 | 390 | rBV2 | 242145 | 440602 | 1.03% | 0.508% |
| 16 | 7.237 | 390 | 393 | 401 | rBV3 | 2255273 | 4982920 | 11.65% | 5.745% |
| 17 | 7.357 | 405 | 408 | 410 | rVV | 6444955 | 6778293 | 15.84% | 7.815% |
| 18 | 7.389 | 410 | 412 | 418 | rVB | 1210448 | 1739108 | 4.06% | 2.005% |
| 19 | 7.461 | 418 | 421 | 427 | rBV2 | 1315172 | 2569041 | 6.00% | 2.962% |
| 20 | 7.525 | 427 | 429 | 432 | rVB2 | 346818 | 440380 | 1.03% | 0.508% |

| | | | | | | | | | | |
|---|---|---|---|---|---|---|---|---|---|---|
| 21 | 7.622 | 438 | 441 | 444 | rBV | 965929 | 1288621 | 3.01% | 1.486% |
| 22 | 7.926 | 472 | 479 | 484 | rBV2 | 475648 | 923233 | 2.16% | 1.064% |
| 23 | 8.287 | 519 | 524 | 535 | rBV3 | 112971 | 574449 | 1.34% | 0.662% |
| 24 | 8.968 | 604 | 609 | 618 | rVV | 451072 | 1330028 | 3.11% | 1.533% |
| 25 | 9.072 | 618 | 622 | 638 | rVV4 | 138540 | 699874 | 1.64% | 0.807% |
| 26 | 9.233 | 638 | 642 | 646 | rVB | 566051 | 715567 | 1.67% | 0.825% |
| 27 | 9.922 | 725 | 728 | 736 | rBV2 | 164635 | 460388 | 1.08% | 0.531% |
| 28 | 14.699 | 1319 | 1324 | 1338 | rVB | 352572 | 838947 | 1.96% | 0.967% |
| 29 | 17.977 | 1718 | 1733 | 1748 | rBV | 15197658 | 42782761 | 100.00% | 49.323% |
| 30 | 18.755 | 1827 | 1830 | 1848 | rVB4 | 111485 | 471113 | 1.10% | 0.543% |

Sum of corrected areas: 86739646

TOXINAT2.M Thu Nov 23 03:31:13 2017
